# Dysbindin Regulates the Transcriptional Level of Myristoylated Alanine-Rich Protein Kinase C Substrate *via* the Interaction with NF-YB in Mice Brain

**DOI:** 10.1371/journal.pone.0008773

**Published:** 2010-01-19

**Authors:** Hiroaki Okuda, Ryusuke Kuwahara, Shinsuke Matsuzaki, Shingo Miyata, Natsuko Kumamoto, Tsuyoshi Hattori, Shoko Shimizu, Kohei Yamada, Keisuke Kawamoto, Ryota Hashimoto, Masatoshi Takeda, Taiichi Katayama, Masaya Tohyama

**Affiliations:** 1 Department of Anatomy and Neuroscience, Graduate School of Medicine, Osaka University, Osaka, Japan; 2 Department of Second Anatomy, Faculty of Medicine, Nara Medical University, Nara, Japan; 3 The Osaka-Hamamatsu Joint Research Center for Child Mental Development, Graduate School of Medicine, Osaka University, Osaka, Japan; 4 Department of Child Development and Molecular Brain Science, United Graduate School of Child Development, Osaka University, Kanazawa University and Hamamatsu University School of Medicine, Osaka, Japan; 5 Department of Psychiatry, Osaka University Graduate School of Medicine, Osaka, Japan; Chiba University Center for Forensic Mental Health, Japan

## Abstract

**Background:**

An accumulating body of evidence suggests that Dtnbp1 (Dysbindin) is a key susceptibility gene for schizophrenia. Using the yeast-two-hybrid screening system, we examined the candidate proteins interacting with Dysbindin and revealed one of these candidates to be the transcription factor NF-YB.

**Methods:**

We employed an immunoprecipitation (IP) assay to demonstrate the Dysbindin-NF-YB interaction. DNA chips were used to screen for altered expression of genes in cells in which Dysbindin or NF-YB was down regulated, while Chromatin IP and Reporter assays were used to confirm the involvement of these genes in transcription of Myristoylated alanine-rich protein kinase C substrate (MARCKS). The sdy mutant mice with a deletion in Dysbindin, which exhibit behavioral abnormalities, and wild-type DBA2J mice were used to investigate MARCKS expression.

**Results:**

We revealed an interaction between Dysbindin and NF-YB. DNA chips showed that MARCKS expression was increased in both Dysbindin knockdown cells and NF-YB knockdown cells, and Chromatin IP revealed interaction of these proteins at the MARCKS promoter region. Reporter assay results suggested functional involvement of the interaction between Dysbindin and NF-YB in MARCKS transcription levels, *via* the CCAAT motif which is a NF-YB binding sequence. MARCKS expression was increased in sdy mutant mice when compared to wild-type mice.

**Conclusions:**

These findings suggest that abnormal expression of MARCKS *via* dysfunction of Dysbindin might cause impairment of neural transmission and abnormal synaptogenesis. Our results should provide new insights into the mechanisms of neuronal development and the pathogenesis of schizophrenia.

## Introduction

Schizophrenia is a common and devastating psychiatric disorder. Lack of patient compliance, due to undesirable side effects and efficacy restricted to positive symptoms, highlights the need to develop novel therapeutics. The etiology of the disease remains unknown, but in recent years a convergence of genetic, pharmacological, and neuroanatomical findings suggest that neural transmission [1]–[4] and synapse formation [5]–[11] are involved in schizophrenia. Recent studies suggest that disturbances of Dysbindin (dystrobrevin-binding protein 1; DTNBP1) are involved in this abnormal neural transmission.

The cause of schizophrenia is thought to involve the combined effects of multiple gene components. Genetic linkage and association studies have identified potential susceptibility genes such as Dysbindin [12], [13], Neuregulin [14], [15], Catechol*-O-*methyltransferase [16]–[18] and RG4 [19]–[22]. In particular, it has been reported that chromosome 6p is one of the highest susceptibility regions in linkage studies of schizophrenia [23], [24]. Among them, genetic variants in a gene 6p22.3 expressing Dysbindin, which is identified as a protein interacting with dystrobevins [25], have been shown to be strongly associated with schizophrenia [12].

In studies on postmortem brain tissue, decreased levels of Dysbindin protein [26] and mRNA [27] have been shown in patients with schizophrenia compared with controls. Chronic treatment of mice with antipsychotics did not affect the expression levels of Dysbindin protein and mRNA in their brains [26], [28], suggesting that evidence of lower levels of Dysbindin protein and mRNA in the postmortem brains of schizophrenics is not likely to be a simple artifact of antemortem drug treatment. In addition, previous reports have shown that diverse high-risk single nucleotide polymorphisms (SNPs) and haplotypes could influence Dysbindin mRNA expression [27], [29]. These data indicate that the Dysbindin gene may confer susceptibility to schizophrenia through reduced Dysbindin expression.

Several lines of evidence suggest that Dysbindin may be associated with brain function. SNPs in *Dysbindin* have been associated with intermediate cognitive phenotypes related to schizophrenia such as IQ and working and episodic memory, and a Dysbindin haplotype has been associated with higher educational attainment [30], [31]. In addition, several reports suggest the involvement of Dysbindin in cognitive functions [32]–[34]. These findings strongly suggest the importance of Dysbindin in brain function. At the cellular level, Dysbindin is located at both pre- and post-synaptic terminals [26], [35], and is thought to be involved in postsynaptic density (PSD) function and the trafficking of receptors (NMDA, GABAergic, and nicotinic). Over-expression of Dysbindin increases glutamate release from pyramidal neurons in cell culture, possibly because of its role in vesicular trafficking [36]. Decreases in Dysbindin mRNA and protein levels have been reported in regions previously implicated in schizophrenia: the prefrontal cortex, midbrain, and hippocampus [26], [27]. However, the molecular mechanisms of how decreases in Dysbindin expression may contribute to vulnerability to schizophrenia remain unknown.

Thus, we examined the interacting partners of Dysbindin using yeast two-hybrid analysis in order to help elucidate the function of Dysbindin. These interacting-protein data suggest that Dysbindin is involved in such processes as neurotransmission, cell signaling, the cytoskeleton and transcription. (Matsuzaki S *et al.* in submission). In addition, our previous reports suggest the following; (1) decreased expression of Dysbindin might increase dopamine release in the brain resulting in the observed abnormal behavior in sdy mice (Dysbindin KO mice) [37], [38], (2) Dysbindin is likely involved in dopaminergic or glutamatergic transmission [36], [39], (3) Dysbindin is likely involved in neurotransmission by binding with the BLOC1 complex, and with transcription by binding with transcription-related genes (Matsuzaki S *et al.* in submission), (4) the expression level of Dysbindin may affect the expression of SNAP25 [36], [39], (5) Dysbindin may play a key role in coordinating JNK signaling and actin cytoskeleton required for neural development [40]. These findings suggest that Dysbindin may influence neurotransmission and neural development *via* interaction with other factors or by regulation of transcription.

In a previous paper, we identified several Dysbindin interacting partners including the transcription factor, nuclear transcription factor Y beta (NF-YB) (Matsuzaki S *et al.* in submission). NF-YB belongs to a family of CCAAT-binding transcription factors, which are important for the basal transcription of a class of regulatory genes and are involved in cellular reactions [41]–[44]. Subsequently, in this study, we examined the functional involvement of Dysbindin in transcription *via* its interaction with NF-YB. As a result, we showed that the NF-YB/Dysbindin complex regulates the transcription of MARCKS *via* interaction with certain CCAAT sequences, and abnormal NF-YB/Dysbindin interaction could cause alterations such as impaired neural transmission and abnormal development of neurons.

## Results

### Dysbindin Exists within the Nucleus in Addition to the Cytoplasm

**Figure 1:**
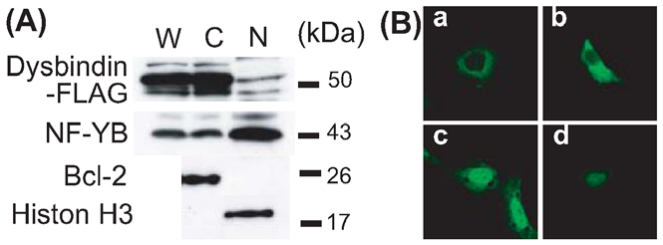
The nuclear localization of Dysbindin. (A) HEK293 cells overexpressing Dysbindin-FLAG were separated into nuclear and cytosolic fractions. Anti-Bcl2 antibody was used for the cytosolic fraction marker and anti-Histone H3 antibody was used for the nuclear fraction marker. W: Whole cell lysates, N: Nuclear Fraction, C: Cytosolic fraction. Dysbindin-FLAG was slightly present in the nuclear fraction. (B) Dysbindin-GFP was overexpressed in HEK293 cells (a and c) or in SH-SY5Y cells (b and d). Dysbindin was usually localized in the cytoplasm and slightly in the nucleus (a and b). After treatment with LMB, a potent inhibitor of CRM1-dependent nuclear export, Dysbindin-GFP accumulated in the nucleus (c and d).

We examined the existence of Dysbindin in the nucleus, because Dysbindin should exist within the nucleus to play a functional role in transcriptional regulation. We used an overexpression vector for Dysbindin tagged with –FLAG or –V5 to check the intracellular localization of Dysbindin. The fractionation study using Dysbindin-FLAG-overexpressing HEK293 cells shows that Dysbindin exists mainly in the cytosol while a small amount exists in the nucleus (Figure 1A), and Dysbindin-V5 showed the same results (data not shown). These results are in accordance with a previous report [45]. Morphologically, Dysbindin is localized mainly in the cytoplasm with a perinuclear high density region in HEK293 cells and SY5Y cells; however, a faint immunoreaction was also seen within the nucleus (Figure 1B -a and -b). Furthermore, pretreatment with leptomycine-B (LPB), which inhibits export from the nucleus to the cytoplasm, caused a slight Dysbindin increase in cells, which then showed nuclear localization of Dysbindin (Figure 1B -c and -d ). These findings suggest that Dysbindin protein is shuttled between the nucleus and the cytoplasm.

### Dysbindin Binds to the Transcription Factor NF-YB

**Figure 2:**
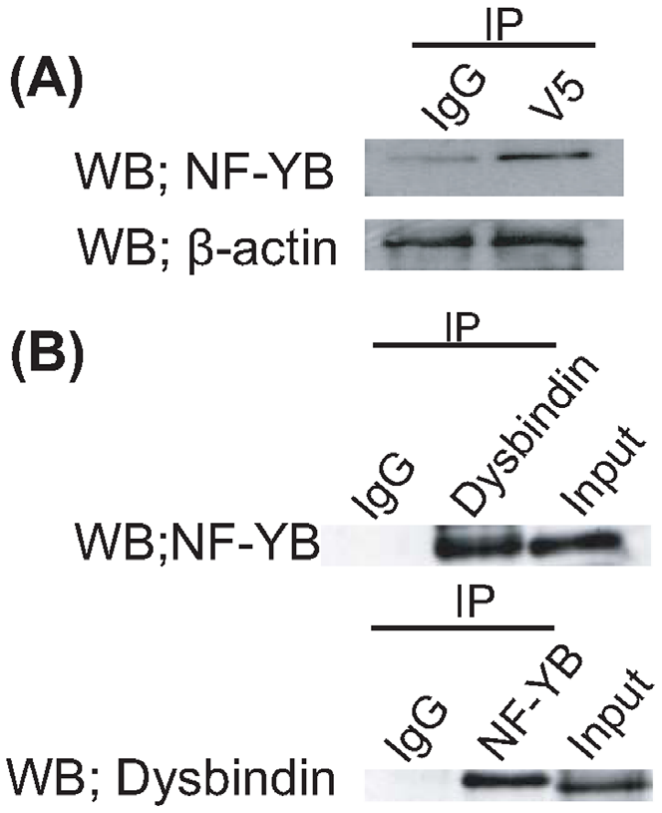
The interaction between Dysbindin and NF-YB. (A) HEK293 cells were transfected with Dysbindin-V5. Immunoprecipitates (IP) of lysates of HEK293 cells expressing Dysbindin-V5 obtained by antibodies to tag proteins (V5) (2^nd^ lane), or nonspecific rabbit IgG (IgG) (1^st^ lane) were subjected to Western blot with anti- NF-YB antibody (upper panel). Dilutions of the lysate (5%, HEK293 cells) were subjected to Western blot with anti-β-actin antibody (lower panel). (**B**) Immunoprecipitates (IP) of lysates of SH-SY5Y cells obtained by antibodies to Dysbindin (upper panel 2^nd^ lane), NF-YB (lower panel 2^nd^ lane), or nonspecific rabbit IgG (IgG) (1^st^ lane of both panels) were subjected to Western blot with anti- NF-YB antibody (upper panel) or Dysbindin antibody (lower panel). Dilutions of the lysate (5%, HEK293 cells) were subjected to Western blot with anti-NF-YB antibody (3^rd^ lane of upper panel) or Dysbindin antibody (3^rd^ lane of lower panel).

Using yeast two-hybrid screening, we identified several transcriptional factors as candidates that may interact with Dysbindin. We selected NF-YB, one of the candidates, and confirmed a Dysbindin-NF-YB interaction by immunoprecipitation assay using HEK293T cells which express NF-YB endogenously (Figure 1A). HEK293T cells were transfected with expression vectors for Dysbindin-V5, and cell lysates were subjected to immunoprecipitation with anti-V5 or anti-NF-YB antibodies, followed by Western blot analysis with a reciprocal antibody. NF-YB was detected in the immunoprecipitates with an anti-V5 body, comparing to the immunoprecipitates with control IgG (Figure 2A), while Dysbindin-V5 was detected in the immunoprecipitates with an anti-NF-YB antibody, comparing to control IgG (data not shown). Thus, Dysbindin and NF-YB are physiologically associated with each other in transfected mammalian cells.

To further our research, we produced a specific anti-Dysbindin antibody with high titer. The antibody detects endogenous Dysbindin in cell and mouse brain samples, though it did not detect any bands corresponding to Dysbindin from the lysates of Dysbindin knockout mouse brain [40]. The existence of endogenous Dysbindin and endogenous NF-YB in lysates from SH-SY5Y cells was confirmed by Western Blot (Figure 2B, 3^rd^ lane of both panels). Immunoprecipitation using the lysates with antibodies for Dysbindin and NF-YB and subsequent Western blot revealed the interaction of endogenous Dysbindin with endogenous NF-YB (Figure 2B, 2^nd^ lane of both panels), and this binding was also confirmed using adult mouse brain lysates (data not shown).

### Downregulation of Dysbindin Causes Upregulation in Expression Levels of Myristoylated Alanine-Rich Protein Kinase C Substrate (MARCKS)

As shown above, we had revealed an interaction between Dysbindin and NF-YB. This result suggests that Dysbindin may be functionally involved in transcription of some genes regulated by NF-YB. We screened for genes displaying altered expression by means of a DNA chip, using RNA extracts from the Dysbindin or NF-YB knockdown human neural cell line, SH-SY5Y. The expression of either *Dysbindin* or *NF-YB* was decreased by the corresponding siRNA for each gene, and the effects of siRNA on Dysbindin or NF-YB were confirmed by Western blot analysis (Figure S1). The genes showing increased expression in the Dysbindin knockdown cells, as well as in the NF-YB knockdown cells, are listed in Table 1A, while those showing decreased expression are listed in Table 1B. Next, using the DANASIS 2.0 system or sequencing of the promoter region, we screened for genes having the CCAAT sequence in the promoter region, because NF-YB is known to bind with high specificity to the CCAAT motif in the promoter region of a variety of genes (Table 1, gene names shown in red). We then focused on three genes; Myristoylated alanine-rich protein kinase C substrate (*MARCKS*) [46]–[48], Phospholipase C beta 4 (*PLCB4*) [49] and Synaptotagmin 1 (*SYT1*) [50], because an accumulating number of reports point to the involvement of impaired neural transmission in the schizophrenia pathology. In addition, we considered the involvement of the genes in psychiatric diseases and we narrow down to MARCKS [51] and SYT1 [52]. Interestingly, a previous report suggests the alteration of SYT1 in schizophrenia patients [52]. The paper shows increase of SYT1 mRNA in younger schizophrenia patients group, while it shows decrease of SYT1 mRNA in older schizophrenia patients. These results suggest the complicated and multiple regulation of SYT1 transcriptional regulation. Thus, we examined the functional involvement of the Dysbindin-NF-YB interaction in *MARCKS* transcription.

**Table 1:** The list of genes altered by Dysbindin as well as NF-YB.

**(A) Upregulated genes**				
**Dysbindin**		**NF-YB**		
**2 h**	**24 h**	**2 h**	**24 h**	**Gene name**
1.343	1.393	1.234	1.409	“Chaperonin containing TCP1, subunit 4 (delta)”
1.344	1.325	1.232	1.352	BCL2-associated athanogene
1.296	1.406	1.485	1.394	***Thymine-DNA glycosylase***
1.315	1.476	1.261	1.295	***Myristoylated alanine-rich protein kinase C substrate***
1.355	1.559	1.430	1.434	Homer homolog 3 (Drosophila)
1.411	1.400	1.368	1.224	Hypothetical protein MGC2749
1.238	2.037	1.368	1.259	***Secretogranin II ( chromograninC)***
**(B) Decreased genes**				
**Dysbindin**		**NF-YB**		
**2 h**	**24 h**	**2 h**	**24 h**	**Gene name**
0.768	0.701	0.762	0.642	***Brain protein 44-like***
0.747	0.649	0.757	0.677	***Jun dimerization protein 2***
0.827	0.643	0.805	0.803	Kinesin family member 3A
0.734	0.790	0.764	0.601	Sarcosine dehydrogenase
0.814	0.698	0.762	0.722	***Phospholipase C, beta 4″***
0.761	0.645	0.670	0.699	***Synaptotagmin I***
0.815	0.518	0.741	0.780	B cell RAG associated protein
0.729	0.634	0.790	0.796	Hypothetical protein FLJ39370
0.763	0.631	0.760	0.668	SEC63-like (S. cerevisiae)
0.813	0.776	0.824	0.811	***ADP-ribosylation-like factor 6 interacting protein 5***
0.732	0.588	0.608	0.749	***Prothymosin, alpha (gene sequence 28)″***
0.693	0.645	0.769	0.787	***Homeodomain interacting protein kinase 3***
0.710	0.711	0.744	0.618	Similar to AV028368 protein
0.772	0.759	0.762	0.682	***Tropomyosin 4***
0.833	0.651	0.819	0.753	Lactate dehydrogenase A

(A) The genes upregulated by the knockdown of Dysbindin that were in common with those upregulated by the knockdown of NF-YB are listed. The genes showed by bold and italic format have the CCAAT motif.

(B) The genes downregulated by the knockdown of Dysbindin that were in common with those downregulated by the knockdown of NF-YB are listed. The genes showed by bold and italic format have the CCAAT motif.

**Figure 3:**
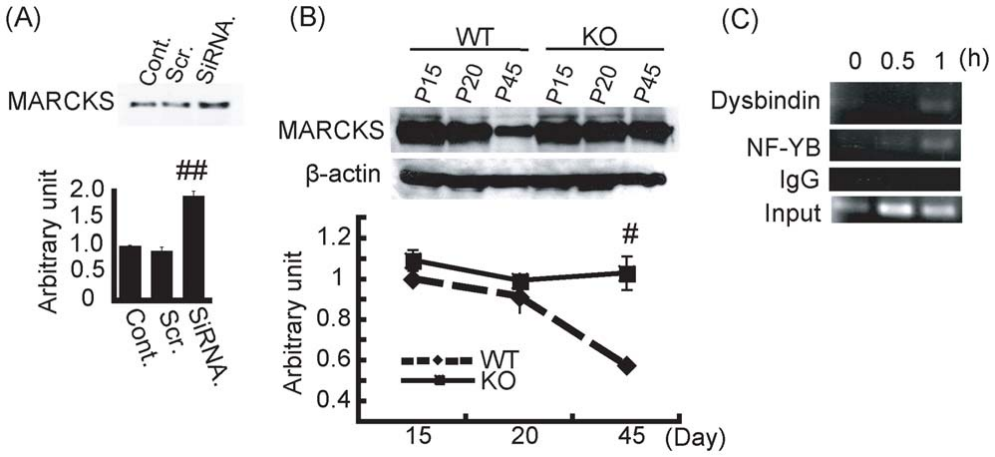
The effects of Dysbindin on MARCKS expression levels. (A) SH-SY5Y cells were transfected with scrambled siRNA or siRNA for Dysbindin. Cell lysate of non-treated cells (Cont.), scrambled RNAi-transfected cells (Scr.) and RNAi for Dysbindin-transfected cells (siRNA) were subjected to Western blot with anti-MARCKS antibody. Columns and vertical bars denote the means ± SEM (triplicate independent experiments). Dysbindin knockdown cells exhibited significant reduction of MARCKS expression compared with control cells (*P*<0.001, Student's t-test). (B) Hippocampus lysates were collected from wild-type mice or Dysbindin KO mice at P15, P20 and P45. The lysates were subjected to Western blot with anti-MARCKS antibody. Graphs and vertical bars denote the means ± SEM (triplicate independent experiments). At P45, Wild-type mice showed significant decreased MARCKS expression, while Dysbindin KO mice showed a maintained MARCKS expression. These data were confirmed by triplicate independent experiments (P<0.01, Student's t-test). (**C**)Chromatin IP (ChIP) was performed using SH-SY5Y cells under the stimulation of retinoic acid. The promoter region of *MARCKS* was detected both in the IPs of anti-Dysbindin antibody (1^st^ panel) and those of anti-NF-YB antibody (2^nd^ panel), but not in the IPs of IgG (3^rd^ panel).

To confirm the involvement of the knockdown of Dysbindin or NF-YB in the upregulation of MARCKS, we performed Western blot analysis using Dysbindin or NF-YB knockdown SH-SY5Ycells. Comparing the expression level of the MARCKS protein with that of control cells, Dysbindin knockdown cells showed upregulation of MARCKS protein (Figure 3A). To confirm the effect of Dysbindin on MARCKS *in vivo*, we examined the expression of MARCKS protein in the hippocampus with advancing age of the Dysbindin knockout mice, comparing with that found in wild-type mice. In the wild-type mice, a peak in MARCKS protein expression in the hippocampus was identified at postnatal day 15 and 20 (Figure 3B), and then decreased markedly over time. However, such a decrease was not observed in the Dysbindin knockout mice, where large amounts of Dysbindin protein were still expressed in the hippocampii of older mice (Figure 3B). These findings suggest that downregulation of Dysbindin may enhance transcription of the *MARCKS* gene, resulting in the upregulation of MARCKS protein.

We performed chromatin IP analysis using SH-SY5Y cells over-expressing Dysbindin-Flag, to explore the possibility that the Dysbindin-NF-YB complex could affect the transcription of *MARCKS via* interaction with the promoter region of *MARCKS*. The cells were stimulated by retinoic acid to induce *MARCKS,* and were collected as the samples for chromatin IP. PCR products from the chromatin IPs suggest that Dysbindin and NF-YB simultaneously interact with the promoter region of *MARCKS*, but control IgG experiments did not show this result (Figure 3C). These findings indicate that the Dysbindin-NF-YB complex interacts with the promoter region of the *MARCKS* gene resulting in inhibition of *MARCKS* transcription.

### The Transcriptional Level of the *MARCKS* Gene Is Regulated by Dysbindin *via* the NF-YB Binding Motif, CCAAT-2

**Figure 4:**
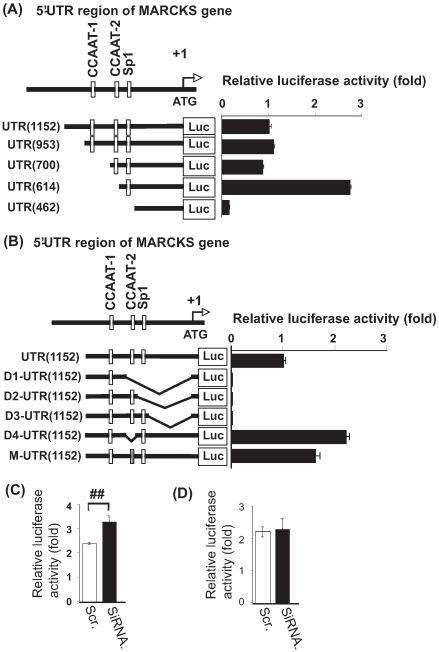
Dysbindin regulates the transcription of MARCKS via the CCAAT2 sequence. (A) The following five vectors were used for luciferase assay, containing shorter DNA probes; UTR(1152)-Luc, UTR (953)-Luc, UTR(700)-Luc, UTR(614)-Luc, and UTR(462)-Luc, were transfected into SH-SY5Y cells and Luciferase activity was measured. UTR(614), which lacks CCAAT1, showed increased luciferase activity. The luciferase activity of UTR(1152) was used as control. Columns and vertical bars denote the means ± SEM (triplicate independent experiments). (B) UTR(1152)-Luc vector and deleted or point mutation of UTR(1152)-Luc vectors, [D1-UTR(1152)-Luc], [D2-UTR(1152)-Luc], [D3-UTR(1152)-Luc] [D4-UTR(1152)-Luc] and [M-UTR(1152)-Luc], were transfected into SH-SY5Y cells and Luciferase activity was measured. [D4-UTR(1152)-Luc], which lacks CCAAT2, and [M-UTR(1152)-Luc], which has a point mutation in the CCAAT2 sequence, showed increased luciferase activity. The luciferase activity of UTR(1152) was used as the control. Columns and vertical bars denote the means ± SEM (triplicate independent experiments). (C and D) Scrambled RNAi-transfected SH-SY5Y cells and Dysbindin RNAi-transfected SH-SY5Y cells were transfected with the UTR(1152)-Luc vector (C) or D4-UTR(1152)-Luc (D) and Luciferase activity was measured. UTR(1152)-Luc vector-expressing cells showed the effect of Dysbindin expression levels on luciferase activity, but D4-UTR(1152)-Luc expressing cells did not. Columns and vertical bars denote the means ± SEM (triplicate independent experiments; *P*<0.001, Student's t-test).

As shown in Figure 4A, the 5′−UTR region of the *MARCKS* gene has two kinds of CCAAT sequences; one CCAAT motif located between UTR −1152 and −700 and the other located between UTR −700 and −614. In this study, we tentatively named the former CCAAT sequence “CCAAT-1” and the latter “CCAAT-2.” It is well known that NF-YB binds to the CCAAT motif to regulate transcription of target genes. Thus, we examined whether CCAAT motifs are essential to the regulation of *MARCKS* transcription by means of a luciferase assay, using the following five vectors containing shorter RNA probes; UTR(1152)-Luc, UTR (953)-Luc, UTR(700)-Luc, UTR(614)-Luc, and UTR(462)-Luc (Figure 4A). These constructs were transiently transfected into SH-SY5Y cells which express Dysbindin and NF-YB endogenously, and luciferase activity in each cell line was measured 24 hours after stimulation with retinoic acid. As baseline, we used luciferase activity detected in the SH-SY5Y cells expressing the UTR(1152)-Luc after retinoic acid stimulation (Figure 4A). In the cells transfected with UTR (953)-Luc containing both CCAAT sequences and UTR(700)-Luc containing the CCAAT-1 sequence but lacking the CCAAT-2 sequence, luciferase activity remained at baseline level after stimulation with retinoic acid (Figure 4A). However, luciferase activity was markedly increased in the cells expressing UTR(614)-Luc after retinoic acid stimulation (Figure 4A). These results suggest that the CCAAT-2 motif plays an important role in inhibition of *MARCKS* transcription. Furthermore, the SH-SY5Y cells transfected with UTR(462)-Luc lacking CCAAT-1, CCAAT-2 and the Sp1 region showed very low luciferase activity (Figure 4A), indicating that Sp1 is indispensable for *MARCKS* transcription.

To confirm that the CCAAT-2 region is important in regulation of *MARCKS* transcription, we prepared several probes for the luciferase assay; D1-UTR(1152)-Luc which lacks the CCAAT-2 motif and its downstream region including Sp1 from UTR(1152)-Luc, D2-UTR(1152)-Luc which lacks the Sp1 region and downstream sequence from UTR(1152)-Luc, D3-UTR(1152)-Luc which lacks only sequence downstream of the Sp1 region, D4-UTR(1152)-Luc which lacks only the CCAAT-2 motif from UTR(1152)-Luc, and M-UTR(1152)-Luc which has a point mutation in the CCAAT-2 motif (Figure 4B). Luciferase activity was detected from the SH-SY5Y cells transfected with each probe, and luciferase activity detected in the cells transfected with UTR(1152)-Luc was used as the baseline value (Figure 4B). Cells transfected with M-UTR(1152)-Luc and those transfected with D4-UTR(1152)-Luc exhibited marked increases in luciferase activity (Figure 4B), showing that the CCAAT-2 motif plays a key role in inhibition of *MARCKS* transcription. Furthermore, cells expressing D1-UTR(1152)-Luc, D2-UTR(1152)-Luc or D3-UTR(1152)-Luc exhibited no luciferase activity. These findings suggest that the sequence downstream of the Sp1 region, as well as the Sp1 region itself, is indispensable for *MARCKS* transcription.

To confirm the involvement of Dysbindin in the altered *MARCKS* transcription levels *via* the CCAAT-2 motif, we compared the luciferase activity of UTR(1152)-Luc detected in Dysbindin knockdown cells with that of control cells. As shown in Figure 4C, knockdown of Dysbindin resulted in upregulation of luciferase activity in the UTR(1152)-Luc transfected cells. However, the effect of knockdown of Dysbindin on luciferase activity was not observed in the D1-UTR(1152)-Luc transfected cells (Figure 4D). These results suggest that Dysbindin regulates *MARCKS* transcription *via* the CCAAT2 motif; the NF-YB binding site. On the other hand, since negligible levels of luciferase activity were observed in cells transfected with any of the probes lacking the sequence downstream of the Sp1 region, the sequence downstream of Sp1 appears to be essential for *MARCKS* transcription (Figure 4A and 4B).

## Discussion

Numerous reports support the role of Dysbindin in the etiology of schizophrenia [13], [30], [37], [53]–[60]. Previous studies have reported a decrease in Dysbindin expression in the brains of schizophrenic patients both at the mRNA and protein levels [26], [27]. However, the functional involvement of Dysbindin in the neural system is not yet well elucidated. In this study, we examined involvement of Dysbindin in neural transmission and neural formation *via* transcriptional regulation, because abnormalities in these neural processes are very important in the pathogenesis of schizophrenia.

### Regulation of *MARCKS* Transcription by the Dysbindin/NF-YB Interaction

As a result of the yeast-two-hybrid assay and immunoprecipitation assay, we revealed an interaction between NF-YB and Dysbindin (Figure 1 and 2). In addition, we showed the binding of NF-YB and Dysbindin to the *MARCKS* promoter region (Figure 3C). These findings suggest involvement of this complex in transcriptional regulation of MARCKS. As shown in Figure 4, we found two CCAAT sequence motifs at the 5′−UTR of the *MARCKS* gene. Previous reports show that members of the NF-Y family including NF-YB bind to CCAAT sequences and can regulate transcription of a number of genes. Our results suggest that one of the CCAAT sequences, CCAAT-2, is important for *MARCKS* transcriptional regulation. On the other hand, our luciferase assay results suggest that both the Sp1 region and the sequence downstream of Sp1 are indispensable for *MARCKS* transcription (Figure 4A and 4B).

### Dysbindin Knockdown Increases MARCKS Protein Levels *In Vivo* and *In Vitro*

In accordance with the enhanced *MARCKS* transcription mediated by the knockdown of Dysbindin, Dysbindin knockdown cells show increased MARCKS levels (Figure 3A). Next, we examined the expression level of MARCKS in Dysbindin knockout mice. As shown in Figure 3b, in the wild-type mouse brain the peak in MARCKS expression is at postnatal day 15; thereafter decreasing markedly with advancing age until only low levels of MARCKS expression are seen in adults (P45). Comparable alternations in MARCKS expression were also observed in another mouse line, ICR (data not shown). These findings support the hypothesis that MARCKS plays an important role in brain development. However, in the Dysbindin knockout mice, there is no effect on MARCKS expression during the developmental stage, when MARCKS is abundantly expressed in wild-type mice. During this stage, *MARCKS* transcription may be regulated by multiple molecules, which compensate for the lack of Dysbindin. With increasing age of the mouse, MARCKS expression decreases gradually to a low level of expression in adults (Figure 3b). In contrast, a decrease in MARCKS expression was not observed in Dysbindin knockout mice (Figure 3b) and even in adult mice brains, a high level of expression of MARCKS was detected. These findings show that Dysbindin likely plays a major role in regulation of MARCKS expression in the adult brain, in contrast to in the developmental stage. Therefore, considering the results in Dysbindin knockout mice, it is likely that MARCKS is expressed at high levels in schizophrenic brains, compared with age-matched control brains.

### MARCKS and Neural Transmission

It has been shown that MARCKS impacts on neurotransmission *via* F-actin and on vesicular transport *via* synaptic vesicles [46]–[48]. Furthermore, many reports indicate that dopaminergic transmission is increased in the brains of schizophrenics [1]–[4]. Dopamine D2 antagonists are an effective treatment in schizophrenia, and dopamine-enhancing drugs mimic psychotic symptoms of schizophrenia. In the schizophrenic brain, the expression of Dysbindin is decreased, resulting in an increase in MARCKS protein expression, which impacts on neurotransmission. Furthermore, we found that decreases in Dysbindin levels upregulate dopamine release [39]. Therefore, the enhanced dopaminergic transmission produced by the lower expression level of Dysbindin may be partially attributable to activation of MARCKS. Thus, the impairment of neural transmission in the schizophrenic brain may be caused by alterations of MARCKS expression levels *via* changes in Dysbindin.

### Dysbindin May Regulate Neural Formation *via* Alteration of MARCKS Levels

Many studies support the hypothesis that schizophrenia is a neurodevelopmental disease. Disrupted-In Schizophrenia 1 (DISC1) is a gene disrupted by a (1;1) (q42.1; q14.3) translocation that segregates with major psychiatric disorders, including schizophrenia in a Scottish family [61], [62]. Previously, we examined the physiological role of the molecular complex composed of DISC1 and its interacting partners, Fasciculation and elongation protein zeta 1 (Fez1) [63] and DISC1-Binding Zinc finger protein (DBZ)[64]. Both the DISC1-Fez1 interaction and the DISC1-DBZ interaction are involved in neurite extension. These reports suggest that abnormalities in the schizophrenia susceptibility genes, such as DISC1, likely cause an impairment of brain development resulting in schizophrenia. In addition, several reports suggest that the PKC signal is involved in psychiatric disorders, as well as other signals such as ERK, which play important roles in neural development. In addition, we previously showed the importance of Dysbindin for growth cone formation [40]. These previous reports suggest that abnormal neural formation could cause psychiatric disorders and that Dysbindin may be one of the important factors in normal neural development. In this study, we demonstrate the transcriptional regulation of MARCKS *via* Dysbindin and the upregulation of MARCKS by downregulation of Dysbindin. Since MARCKS is involved not only in neural transmission^48^ but also in neural developmental processes such as synaptogenesis and maintaining spine morphology [46], [47], these results suggest that dysfunction of Dysbindin likely causes the upregulation of MARCKS and may induce abnormal development of the nervous system *via* alterations of MARCKS levels.

Thus, in this paper, we report the following findings; (1) Dysbindin interacts with NF-YB, (2) NF-YB and Dysbindin bind to the promoter region of MARCKS, (3) one of the CCAAT sequences is likely essential for the transcriptional regulation of MARCKS and (4) the downregulation of Dysbindin upregulates the expression of MARCKS *in vitro* and *in vivo*. On the other hand, we previously showed that Dysbindin knockout mice exhibit schizophrenia-like behavior and abnormalities of the dopaminergic system. These phenotypes may be at least partly attributable to over-activation of MARCKS *via* a decrease in Dysbindin levels.

In conclusion, these results may help shed some light on the causes of schizophrenia, and indicate that the transcriptional regulation of Dysbindin may contribute to schizophrenia. Further studies of Dysbindin and its association with MARCKS and with schizophrenia may reveal novel treatment targets for schizophrenia.

## Materials and Methods

### Antibodies

Monoclonal anti-Dysbindin antibody was produced. Briefly, GST-fused human Dysbindin was used as antigen and the Dysbindin protein for ELISA was made by thrombin digestion of GST-Dysbindin. High-titer clones for Dysbindin were selected by ELISA using the Dysbindin protein and the immunoreactivity of the clones was checked by Western blot. Antibodies of anti-GFP (Santa Cruz Biotechnology, Santa Cruz, CA), anti-Flag (Sigma-Aldrich, St Louis, MO), anti-V5 (Invitrogen), anti-β-actin (Chemicon International, Temecula, CA), anti-NF-YB (Santa Cruz Biotechnology), anti-MARCKS (Upstate), HRP-conjugated anti-mouse and Rabbit IgG (Cell Signaling Technology, Beverly, MA), and mouse normal IgG (Sigma-Aldrich) were purchased commercially.

### Plasmids

We previously constructed the pEGFP-C1 expression vector (Clontech) carrying the full-length human *Dysbindin* cDNA (-GFP is tagged to N-terminal) [22]. The human Dysbindin-V5 (-V5 is tagged to C-terminal), Dysbindin-FLAG (-FLAG is tagged to N-terminal) and NF-YB moieties were amplified from a human brain cDNA library using PCR and subcloned into pcDNA3.1 (+) expression vector (Invitrogen, Carlsbad, CA). Dysbindin and NF-YB were amplified using rTaq DNA polymerase (Takara Bio Inc., Kyoto, Japan) with the following primer set: Dysbindin-V5, 5′−CTCGAGTTACGTAGAATCGAGACCGAGGAGAGGGTTAGGGATAGGCTTACCAGAGTCGCTGTCCTCACC−3′ (forward) and 5′−GGTACCGCCACCATGCTGGAGACCCTTCGCGA−3′ (reverse); NF-YB, 5′−GCTAGCGCCACCATGACAATGGATGGTGACAGTTCT−3′ (forward) and 5′−GATATCTGAAAACTGAATTTGCTGAAC−3′ (reverse). The amplified fragments were TA cloned into the pGEM-T vector (Promega Corp.).

pMARCKS-Luc(−1152) was generated by subcloning promoters into pGL3-(R2.2) Basic (Promega). We generated 5′ deletion constructs of pMARCKS-Luc(−1152) and an internal deletion construct of the region −700∼−1. Other deletion constructs of the region (−231∼−150) and point mutation constructs of pMARCKS-Luc/dl(−204∼−187), were generated by inserting double-stranded oligonucleotides (Figure 2B and 2D). The plasmid pMARCKS-Luc(−736/mt) was generated by site-directed mutagenesis, which changed the same nucleotides as those of mutant 5.

### Cell Culture

Human neuroblastoma SH-SY5Y cells were obtained from the Human Science Research Resources Bank (HSRRB). These cells were maintained in tissue culture dishes (Nalge Nunc, Rochester, NY, USA) in 50% minimal essential medium (Invitrogen) /50% F-12 (Invitrogen) containing 15% heat-inactivated fetal bovine serum (Invitrogen) at 37°C in an atmosphere of 95% air /5% CO_2_.

### Animals

*sdy* mice (Dysbindin KO mice) and wild-type littermates were provided by the Takeda lab, Department of Psychiatry, Osaka University Graduate School of Medicine. The mice were deeply anesthetized with sodium pentobarbital. Brains (hippocampus) were dissected from each aged mouse. All animal experiments were carried out in accordance with a protocol approved by the Institutional Animal Care and Use Committee of Osaka University.

### Immunocytochemistry

SY5Y cells were grown on poly-l-lysine-coated four-well chamber dishes at a density of 3×10^4^ cells/cm^2^. The cells were fixed in 2% paraformaldehyde in 0.1 M PBS, permeabilized, and blocked with 0.02 M PBS containing 0.3% Triton X-100, 3% BSA and 10% goat serum for 30 min at room temperature, and then incubated with antibodies specific for the individual protein. Confocal microscopy was performed using a Carl Zeiss LSM-510 confocal microscope.

### Fractionation Assay

Cells were collected after washing with ice-cold PBS. Cells and brains were homogenized in Tris buffer (20 mM Tris–HCl, pH 7.8, 1 mM EDTA, 150 mM NaCl and protease inhibitor cocktail (Roche)). After homogenization, the homogenized proteins were lysed by the addition of 0.5% NP-40 for 30 min on ice and centrifuged at 500 ×*g* for 10 min to collect the nuclear pellet. The supernatant was collected as the cytosolic fraction.

### Immunoprecipitation (IP)

After washing cells with ice-cold PBS, cells were collected and resuspended in 1 mL lysis buffer (20 mM Tris–HCl, pH 7.8, 0.2% NP-40, 1 mM EDTA, 150 mM NaCl and protease inhibitor cocktail (Roche)). Cells were frozen in dry ice/EtOH and stored at −80°C. Cell lysates were incubated on ice for 30 min and then centrifuged for 5 min at 13,600×*g*. After centrifugation, the supernatants were precleared with protein Sepharose G beads and IP was carried out in lysis buffer with antibody/protein G Sepharose beads for 1 h at 4°C. After washing in lysis buffer, immunoprecipitated proteins were immunoblotted.

### Immunoblotting

Aliquots of whole cell lysates or IP lysates separated by SDS-PAGE were blotted onto an Immobilon-P membrane (Millipore), and then incubated with antibodies specific for individual protein. Proteins were detected by ECL plus Western Blotting Detection System (GE Healthcare), followed by exposure to X-ray films according to the manufacturer's protocol.

### Knockdown Experiment Using Small Interfering RNA (siRNA)

Stealth siRNA against Dysbindin (5′−CCAAAGUACUCUGCUGGAUUAGAAU−3′ and 5′−GCUCCCAGCUUUAAUCGCAGACUUA−3′ ), NF-YB 5′−UACUGAGGACAGCAUGAAUGAUCAU−3′ , and negative control duplexes (scrambled siRNA for Dysbindin, 5′−CCATGATCTCGTCGTTAGAAAGAAA−3′ and 5′−GCTACCGTTATTAGCACAGCCCTTA−3′ ; and scrambled siRNA for NF-YB, 5′−UACGGAACAACGAGUGUAUAUGCAU−3′ ) were provided by Invitrogen Corp. SY5Y cells were transfected with 100 pM of each siRNA and scrambled siRNA using Lipofectamine 2000 (Invitrogen Corp.) according to the manufacturer's instructions.

### RNA Extracts and Microarray

Total RNA was extracted from cells using RNeasy columns (Qiagen) according to the manufacturer's instructions. Five hundred nanograms of total RNA from control and experimental cells was separately amplified and labeled with either Cy3- or Cy5-labeled CTP (Perkin Elmer) with an Agilent low input linear amplification kit (Agilent Technologies) according to manufacturer's instructions. After labeling and cleanup, amplified RNA was quantified by UV-vis spectroscopy. One microgram each of Cy3- and Cy5-labeled targets were combined and hybridized with a Whole Human Genome Oligo Microarray Kit (G4112F)according to the manufacturer's instructions. Three biological replicates were used at each time point with one of the replicates being a dye reversal of the other two. Microarrays were imaged on a Hitachi image scanner and data analyzed with GeneSpring 6 (Silicon Genetics).

### Chromatin Immunoprecipitation (ChIP) Assay

ChIP analysis was performed using a Chromatin Immunoprecipitation Assay Kit (Upstate Biotechnology) according to the manufacturer's instructions. Briefly, protein−DNA complexes were crosslinked with 1% formaldehyde (10 min at room temperature) and cells were harvested. DNA was sonicated to lengths of 500−1000 bp. Antibodies specific for individual protein were used for immunoprecipitating protein-DNA complexes overnight at 4°C. PCR was performed with individual specific primer sets for the MARCKS promoter: the proximal CCAAT region, 5′−GGTTTGCTCTTTGATGCTCTTGAT−3′ and 5′−ACTTTCGGGTGGGGTGTAA−3′

### Reporter Assay

Reporter plasmids were transfected into cells using Lipofectamine 2000 (Invitrogen) together with phRG-TK (Renilla reporter for internal control) which monitored transfection efficiency. Luciferase activities were assayed using the Dual Luciferase Assay System (Promega). All assays were performed three times in duplicate and values are shown as means ± SD.

## Supporting Information

Figure S1 The preparation of mRNAs for microarray analysis. (A-(a) and B-(a)) To prepare RNAs for microarrays analysis, we transfected the siRNA for Dysbindin, NF-YB, or scrambled as a control. The effect of each RNAi was confirmed by Western blot using the antibody for Dysbindin or NF-YB. (A-(b) and B-(b)) The columns and vertical bars denote the means ± SEM (triplicate independent experiments; P<0.001, Student's t-test). Dysbindin or NF-YB was knocked-down significantly by transfection of the siRNA for Dysbindin or NF-YB, compared with the control.
(1.10 MB EPS)
